# Ultrastructural Analysis of Chikungunya Virus Dissemination from the Midgut of the Yellow Fever Mosquito, *Aedes aegypti*

**DOI:** 10.3390/v10100571

**Published:** 2018-10-18

**Authors:** Asher M. Kantor, DeAna G. Grant, Velmurugan Balaraman, Tommi A. White, Alexander W. E. Franz

**Affiliations:** 1 Department of Veterinary Pathobiology, University of Missouri, Columbia, MO 65211, USA; amkt33@missouri.edu (A.M.K.); balarama@vet.k-state.edu (V.B.); 2 Electron Microscopy Core Facility, University of Missouri, Columbia, MO 65211, USA; GrantDe@missouri.edu (D.G.G.); whiteto@missouri.edu (T.A.W.); 3 Department of Biochemistry, University of Missouri, Columbia, MO 65211, USA

**Keywords:** chikungunya virus, mosquito, midgut, dissemination, ultrastructural, FIB-SEM, electron microscopy, Mayaro virus, bloodmeal, basal lamina

## Abstract

The transmission cycle of chikungunya virus (CHIKV) requires that mosquito vectors get persistently infected with the virus, following its oral acqsuisition from a vertebrate host. The mosquito midgut is the initial organ that gets infected with orally acquired CHIKV. Following its replication in the midgut epithelium, the virus exits the midgut and infects secondary tissues including the salivary glands before being transmitted to another host. Here, we investigate the pattern of CHIKV dissemination from the midgut of *Aedes aegypti* at the ultrastructural level. Bloodmeal ingestion caused overstretching of the midgut basal lamina (BL), which was disrupted in areas adjacent to muscles surrounding the midgut as shown by scanning electron microscopy (SEM). Using both transmission electron microscopy (TEM) and focused ion beam scanning electron microscopy (FIB-SEM) to analyze midgut preparations, mature chikungunya (CHIK) virions were found accumulating at the BL and within strands of the BL at 24–32 h post-infectious bloodmeal (pibm). From 48 h pibm onwards, virions no longer congregated at the BL and became dispersed throughout the basal labyrinth of the epithelial cells. Ingestion of a subsequent, non-infectious bloodmeal caused mature virions to congregate again at the midgut BL. Our study suggests that CHIKV needs a single replication cycle in the midgut epithelium before mature virions directly traverse the midgut BL during a relatively narrow time window, within 48 h pibm.

## 1. Introduction

Chikungunya virus (CHIKV) is an Old World alphavirus (family: *Togaviridae*) belonging to the Semliki Forest virus sero-complex [1]. Originating from Africa, the virus is also highly prevalent in Southeast Asia, including the Indian subcontinent [2,3]. At the end of 2013, CHIKV emerged in the Western Hemisphere, initially in the Caribbean followed by outbreaks in Brazil [4,5]. Meanwhile, the virus has been reported in most countries of South and Central America, including sporadic incidences in the southern United States. Typical chikungunya (CHIK) disease symptoms in humans include febrile illness, rash, myalgia, joint swelling, and arthralgia, which can be long-lasting [3]. Like all other alphaviruses, CHIKV is mosquito-borne, with *Aedes aegypti* and *Ae. albopictus* functioning as the principal CHIKV vectors in urban transmission cycles. 

A mosquito needs to be persistently infected with an arbovirus, such as CHIKV, in order to transmit the virus to susceptible hosts. The mosquito midgut consists of a single layer of epithelial cells surrounded at the basal side by a multi-stranded basal lamina (BL) predominantly consisting of laminin and collagen IV [6,7,8]. The anterior portion of the midgut takes part in sugar absorption whereas the posterior region is responsible for bloodmeal digestion. Following the ingestion of a viremic bloodmeal from a CHIKV infected human, the virus enters the lumen of the mosquito midgut wherein the bloodmeal is digested. Before the bloodmeal is surrounded by the peritrophic matrix, virions need to enter the midgut epithelial cells via receptor-mediated endocytosis [8,9]. At the ultrastructural level, the following zones can be distinguished in a cross section through a midgut epithelial cell: Prominent microvilli for nutrient uptake at the luminal side, an endoplasmic reticulum (ER) surrounding the nucleus, and a prominent basal labyrinth, which is a network of intracellular spaces located towards the cell’s basal side adjacent to the BL [6,7,8,10,11].

During entry, the viral core is released into the cytoplasm of the epithelial cell and gets disassembled at the ER to release the viral RNA genome for translation [12]. The 5′ two-third portion of the ~12 kb CHIKV positive sense RNA genome encodes a polyprotein (P1234) containing the nonstructural proteins nsP1, nsP2, nsP3, and nsP4. Early during viral replication, minus-strand RNA is synthesized from the viral genome based on cleavage of nsP4 from the P1234 polypeptide. Cleavage of P123 into nsP1, nsP2, and nsP3 facilitates plus-strand (=genomic) RNA synthesis. In a later step, 26S RNAs are produced from a subgenomic promoter present on the minus-strand RNA [13]. Translation of the subgenomic RNA results in the expression of the structural proteins, C, PE2, 6K/TF, and E1. In the cytoplasm, *de novo* synthesized genomic RNA and C (capsid) proteins assemble to form the nucleocapsid. The glycoproteins PE2 (including E2 and E3) and PE1 are translocated across the ER and then processed and transported though the Golgi network to the plasma membrane. There, the nucleocapsid associates with the glycoproteins, resulting in budding of the mature virion, which now possesses 80 spikes consisting of trimers of E2 and E1 heterodimers. *De novo* synthesized virions then need to exit the midgut epithelium in order to disseminate to secondary tissues, such as fat body, nerve tissue, hemocytes, and the salivary glands [14,15,16]. Once these tissues are productively infected, virions are released in the saliva, which is injected by the mosquito into the vertebrate host during probing or bloodfeeding. 

The mechanism underlying the viral exit from the midgut is not well understood [8,17,18]. Most of the previous ultrastructural studies in which arbovirus dissemination from the midgut was investigated were conducted with *Culex* spp., which had been infected with flaviviruses, alphaviruses, or bunyaviruses [19,20,21,22,23,24,25]. Flaviviruses, such as St. Louis encephalitis virus (SLEV) or West Nile virus (WNV), and bunyaviruses, such as Rift Valley fever virus (RVFV), employ different replication and virion assembly strategies in an infected cell than alphaviruses [26,27]. Earlier observations also suggested that alphaviruses in general disseminate more rapidly from the mosquito midgut than flaviviruses or bunyaviruses [22,28]. Other ultrastructural studies on alphavirus mosquito infections did not focus on the viral dissemination mechanism [29,30,31,32,33,34,35]. Apart from exiting the posterior midgut through the BL, several studies describe that arboviruses also infect the anterior midgut region, including the intussuscepted foregut/cardia [14,21,36,37,38,39,40]. However, the role of intussuscepted foregut/cardia infection in virus dissemination to secondary tissues is not clear [8] and so far, infection of these tissues with CHIKV has not been reported.

Numerous reports describe the phenomenon of a midgut escape barrier causing virus dissemination from the mosquito midgut to be inefficient or delayed although the midgut epithelium is strongly infected [20,41,42,43,44,45]. Furthermore, the BL surrounding the midgut has been proposed as the principal barrier to arbovirus dissemination, including that of CHIKV [17,18,46]. The pore size exclusion limit for the BL of a mosquito midgut has been estimated to be just 9–12 nm, whereas CHIK virions have a diameter of 60–70 nm [9,11,17,47,48]. Thus, in order for virions to pass through the BL, its pore size exclusion limit needs to be (at least temporarily) enlarged. Recently, we investigated the role of matrix-metalloproteinases (MMPs) as a group of enzymes potentially involved in BL remodeling during bloodmeal digestion and CHIKV dissemination from the mosquito midgut [18,46,49]. Our group has also demonstrated the principal barrier character of the BL to CHIKV. Intrathoracically injected virus was plausibly unable to infect the midgut of sugarfed mosquitoes and virions therefore accumulated at the basal (hemocoel) side of the midgut-surrounding BL [18]. The female mosquito deposits an ingested sugarmeal into her crop instead of directing it immediately to the midgut. However, intrathoracically injected CHIK virions were readily able to infect the midgut epithelium when there was a bloodmeal present in the midgut lumen. Furthermore, substituting a bloodmeal for a protein (bovine serum albumin) or saline (phosphate buffer saline) meal did not negatively affect the ability of orally acquired CHIKV to disseminate from the midgut [46]. Taken together, these observations allow the conclusion that stretching of the gut tissue may be the critical factor causing the BL to become permissive for the virus. 

The purpose of this study was to closely observe the dissemination pattern of CHIKV from the midgut over time at the ultrastructural level. We used scanning electron microscopy (SEM) to visualize to what extent bloodmeal ingestion caused overstretching and damage to the midgut BL. We conducted a transmission electron microscopy (TEM) based time course study to reveal whether or not CHIK virions continuously disseminated from the midgut and which route of dissemination the virus utilized in *Ae. aegypti*. Using a focused ion beam scanning electron microscope (FIB-SEM) to perform three-dimensional tomography [50,51,52,53,54], we visually confirmed the midgut escape route of CHIKV. We investigated the effect of a consecutive (non-infectious) bloodmeal on the dissemination of CHIKV, which had been orally acquired along with an initial bloodmeal. The observed dissemination pattern of the virus was molecularly confirmed using a highly sensitive viral RNA strand-specific qRT-PCR assay in which we included Mayaro virus (MAYV) to compare its replication dynamics in mosquito tissues with those of CHIKV. MAYV is another alphavirus of the Semliki Forest virus sero-complex resembling CHIKV in terms of clinical disease symptoms in humans and vector interaction with *Ae. aegypti* under laboratory conditions [55]. MAYV is prevalent in the Western Hemisphere, circulating in sylvatic cycles in rain forest areas of Central- and South America. So far, a CHIKV-like urban disease cycle involving *Ae. aegypti* has not been reported for MAYV [56]. 

## 2. Materials and Methods

### 2.1. Mosquitoes

*Ae. aegypti* Higgs White Eye (HWE) mosquitoes, a laboratory strain with an eye-pigment deficiency were maintained at 28 °C with 75–80% relative humidity. For colony maintenance, mosquitoes were exposed to artificial bloodmeals consisting of undiluted defibrinated sheep blood. In between bloodmeals, mosquitoes were fed on raisins.

### 2.2. Infection of HWE Mosquitoes with Viruses

Propagation of CHIKV (strain: 37997) and oral infection of mosquitoes via an artificial bloodmeal have been previously described [18,49,57]. The type strain of MAYV, TRVL 4675, was isolated in 1954 from a human patient in Trinidad [58]. The virus was acquired from the World Reference Center for Emerging Viruses and Arboviruses of the University of Texas Medical Branch, Galveston, TX, USA. Similar to CHIKV, the low-passage MAYV strain was propagated in Vero cells in T-25 flasks at a multiplicity of infection (MOI) of 0.01 using Dulbecco’s Modified Eagle Medium (DMEM) supplemented with 7% fetal bovine serum (FBS). To prepare a MAYV-containing, artificial bloodmeal, cell culture media of infected cells was collected at 32–39 h post-infection (pi) and mixed with defibrinated sheep blood at a ratio of 1:1 before being supplemented with ATP to a final concentration of 1 mM. Using glass feeders (one feeder per mosquito carton), seven day-old HWE mosquitoes were fed for 1 h with the virus containing bloodmeal mixture at 37 °C. Fully engorged females were selected and maintained for subsequent experiments. Midguts and carcass of those females were dissected at various time points post-infectious bloodmeal (pibm). All virus work was performed in a BSL3 facility within the Laboratory of Infectious Disease Research (LIDR) at the University of Missouri.

### 2.3. Generation of cDNA Templates

Mosquitoes orally infected with CHIKV or MAYV were dissected to separate midguts from their carcasses at 8, 24, 32, 48, 72, and 96 h pibm. Total RNA was extracted from mosquito midguts (10 midguts per pool) or carcasses (three carcasses per pool) using TRIzol reagent (Life Technologies, Carlsbad, CA, USA). Total RNA (1 μg) was reverse transcribed using the SuperScript III First-Strand Synthesis System (Life Technologies). The reaction components and thermal conditions were the following: 1 μg RNA was mixed with 2 μL of virus- and strand specific primer (10 pmol/ μL) (Table S1) and 2 μL of dNTP (10 mM) before being incubated at 65 °C for 5 min and snap cooled on ice. The heat denatured RNA was mixed with 4 μL of 5× RT buffer, 1 μL of 0.1 M DTT, 1 μL of reverse transcriptase (15 U/μL), and nuclease-free water to a final volume of 20 μL. The reaction mixture was incubated at 55 °C for 60 min and inactivated at 85 °C for 5 min. 

### 2.4. Taqman qPCR Assays for the Virus-Specific Detection of Plus- and Minus-Strand RNAs

Probes were custom-designed by Integrated DNA Technologies (Coralville, IA, USA) to detect viral plus- and minus-strand RNAs via Taqman q(uantitative)PCR. The Taqman probes contained a 5′ FAM reporter and two quenchers, ZEN and IABkFQ, located in the center and at the 3′ end of the molecule, respectively. The ZEN quencher aided in reducing background, and sensitivity of the probe. The oligonucleotide primers contained 5′ AT-rich flaps (5′AATAAATCATAA3′) to increase detection specificity (Table S1). The qPCR amplification was performed using the iTaq Universal Probes Supermix (Bio-Rad, Hercules, CA, USA). Each reaction had a total volume of 20 μL consisting of 10 μL of 2× master mix, forward and reverse primers at a concentration of 900 nM, 2 μL of cDNA template, and the Taqman probe at a concentration of 250 nM. Amplification reactions were performed for 40 cycles under the following thermo-cycling conditions: Initial denaturation at 95 °C for 2 min, annealing at 60 °C for 30 s, and extension at 60 °C for 30 s. The results were analyzed using standard curves, which were generated for the detection of CHIKV and MAYV viral RNAs by cloning cDNA segments of each virus containing the sequences of the qPCR primers and the probe into the pCR-2.1-TOPO TA vector (Life Technologies). DNA plasmid copy numbers were calculated based on their lengths and concentrations. For each qPCR assay, a new 10-fold dilution series of the standard curve was generated. Each sample consisted of three independent biological replicates.

### 2.5. Midgut Sample Preparation for SEM 

Unless otherwise stated, all reagents were purchased from Electron Microscopy Sciences (Hatfield, PA, USA) and all specimen preparation was performed at the Electron Microscopy Core Facility, University of Missouri. Midguts were dissected from HWE mosquitoes and fixed in 2% (v/v) paraformaldehyde, 2% (v/v) glutaraldehyde containing 100 mM sodium cacodylate buffer, pH 7.35. Fixed tissues were rinsed with 100 mM sodium cacodylate buffer, pH 7.35 containing 130 mM sucrose and further rinsed with water. Secondary fixation was performed using 1% osmium tetroxide (Ted Pella, Redding, CA, USA) in cacodylate buffer followed by treatment in a Pelco Biowave (Ted Pella) operated at 100 Watts for 1 min. Specimens were next incubated at 4 °C for 1 h, then rinsed with cacodylate buffer and thereafter with distilled water. Using the Pelco Biowave, a graded dehydration series (100 Watts for 40 s per exchange) was performed using ethanol. Samples were dried using the Tousimis Autosamdri 815 critical point dryer (Tousimis, Rockville, MD, USA), and then sputter-coated with 10 nm of platinum using the EMS 150T-ES Sputter Coater. Images were acquired with a FEI Quanta 600F environmental scanning electron microscope (FEI, Hillsboro, OR, USA).

### 2.6. Midgut Sample Preparation for TEM

Mosquito midguts were dissected and fixed in 100 mM sodium cacodylate buffer, pH 7.35 supplemented with 2% (v/v) paraformaldehyde and 2% (v/v) glutaraldehyde. Each midgut sample was oriented and suspended in HistoGel (Thermo Scientific, Kalamazoo, MI, USA). Next, samples were rinsed with 100 mM sodium cacodylate buffer, pH 7.35 (Sigma Aldrich, St. Louis, MO, USA) containing 130 mM sucrose. Secondary fixation of the sample was conducted in a Pelco Biowave operated at 100 Watts for 1 min using a 100 mM sodium cacodylate buffer supplemented with 1% osmium tetroxide. Specimens were incubated at 4 °C for 1 h, then rinsed with cacodylate buffer followed by distilled water. *En bloc* staining was performed using 1% aqueous uranyle acetate and incubation at 4 °C overnight followed by rinsing with distilled water. Using the Pelco Biowave, a graded dehydration series (100 Watts for 40 s per exchange) was performed in which ethanol was initially used followed by transition to acetone before dehydrated specimens were finally infiltrated with Epon resin (250 Watt for 3 min) and polymerized at 60 °C overnight. Sample sections were cut to a thickness of 85 nm using an ultra-microtome (Ultracut UCT, EM UC7, Leica Microsystems, Wetzlar, Germany) equipped with a diamond knife (Diatome, Hatfield, PA, USA). Images were acquired with a JEOL JEM 1400 transmission electron microscope (JEOL, Peabody, MA, USA) at 80 kV using a Gatan Ultrascan 1000 CCD camera (Gatan, Pleasanton, CA, USA).

### 2.7. Analyzing CHIK Virion Density in Midgut Epithelial Cells and Measuring Midgut BL Thickness in TEM Images 

In each TEM micrograph, CHIK virions were counted and the surface area of the tissue containing virions was determined based upon the pixel size of each image using the software Gatan Microscopy Suite (Gatan). At least 18 TEM micrographs from three different midgut preparations were analyzed per time point. Thickness of the intact midgut BL was measured in several locations of three different midguts per time point. Measurements were conducted in zones absent of muscle tissue where the BL was often spliced and disorganized. BL measurements were based on the pixel size of each image as analyzed with the Gatan Microscopy Suite. 

### 2.8. Midgut Sample Preparation for Serial Block Face Imaging via FIB-SEM 

Midgut samples were prepared following a modified version of the NCMIR protocols for three-dimensional EM with changes to the sample mounting procedure [59,60]. Primary midgut sample fixation was carried out as described above for SEM and TEM sample preparations. Secondary sample fixation was performed using equal parts 4% (w/v) osmium tetroxide (Ted Pella) and 3% (w/v) potassium ferrocyanide in 100 mM cacodylate buffer, pH 7.35 followed by treatment in a Pelco Biowave operated at 100 Watts for 1 min. Specimens were incubated on ice for 1 h, then first rinsed with cacodylate buffer and then with distilled water. Samples were then incubated at room temperature for 1 h in a 1% (w/v) thiocarbohydrazide solution followed by distilled water rinses. Rinsed tissues were incubated in an additional 2% (w/v) aqueous osmium tetroxide solution for 30 min at room temperature, then rinsed again with distilled water. Initial *en bloc* staining was performed using 1% (w/v) aqueous uranyl acetate and incubation at 4 °C overnight followed by a finale rinse with distilled water. Additional *en bloc* staining was performed using Walton’s lead aspartate solution for 30 min at 60 °C followed by additional distilled water rinses. Using the Pelco Biowave, a graded dehydration series (per exchange, 100 Watts for 40 s) was performed using ethanol followed by transitioning into acetone. Dehydrated tissues were then infiltrated with Durcupan ACM resin (250 Watt for 3 min) and polymerized at 60 °C overnight. All samples were trimmed and sectioned to a thickness of 85 nm to be initially examined by TEM (JEOL JEM 1400 operated at 80 kV). This allowed for precise identification of virus-infected regions in the tissue and measurements were taken using landmarks that could be easily identified in the FIB-SEM. To ensure all FIB-SEM data were collected in a cross section, the tissue was mounted with the block face perpendicular to the stub surface. The area of interest was identified using the landmarks established in TEM and a 2-μm layer of platinum was deposited on the block face using the ion column. Next, the area of interest was polished using an ion beam setting of 30 kV, 1 nA and 20-nm sections were subsequently removed from the sample using ion beam settings of 30 kV, 1 nA. FIB-SEM data was collected using a FEI Scios Analytical Dualbeam SEM operated at 2 kV with 0.2 nA current and equipped with a T1-BSE detector to capture the sample images. Using the Amira software suite v. 5.3.1 (FEI Visualization Science Group, Hillsboro, OR, USA), the recorded stack of 108 images from the sample was aligned and segmented to render the three-dimensional model. Based on the data obtained with the Amira software, the model was then graphically refined in Maxon Cinema 4D R19 (MAXON Computer GmbH, Friedrichsdorf, Germany) using the software OTOY Octane Render V3 (OTOY Inc., Los Angeles, CA, USA). 

## 3. Results

### 3.1. Bloodmeal Ingestion Causes Temporal Midgut Tissue Expansion and Damage to the BL

**Figure 1:**
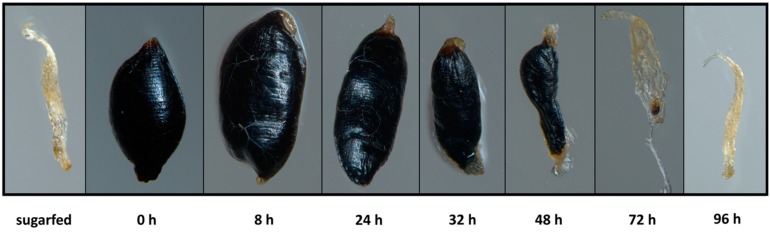
Visual comparison of bloodmeal digestion over time in the midgut of *Aedes aegypti*. One week-old Higgs White Eye (HWE) females ingested an artificial bloodmeal consisting of undiluted defibrinated sheep blood. Light microscope magnification: 30×.

In laboratory maintained HWE mosquito females, a pure bloodmeal consisting of undiluted defibrinated sheep blood was digested within 3 days post-oral acquisition via membrane feeding (Figure 1). As shown in Table S2, the midgut tissue of a fully engorged female was 20× expanded based on an average increase in volume from 0.2 μL (midgut of a sugarfed female) to 3.9 μL (midgut of a bloodfed female). As a consequence of nutrient absorption and desiccation, the volume of the bloodmeal gradually shrank until almost completely digested at 72 h post-(non-infectious) bloodmeal (pbm) (Figure 1). Concurrently, the overstretched midgut tissue contracted until the pre-bloodfeeding condition was reached again between 72 and 96 h pbm. 

**Figure 2:**
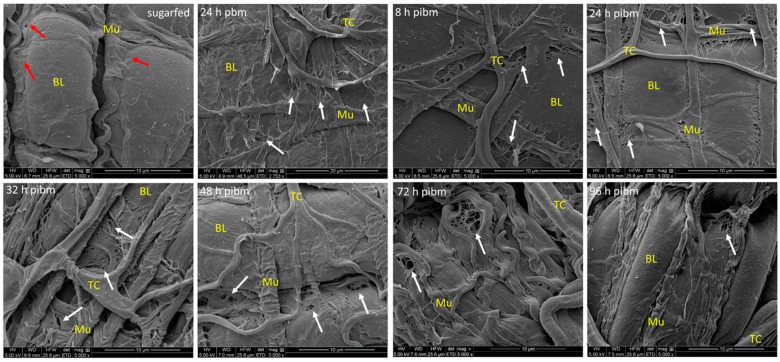
Ultrastructural (SEM) imaging of the midgut surface of sugarfed *Ae. aegypti* in comparison to mosquitoes that had ingested a non-infectious, artificial bloodmeal at 24 h post-bloodmeal (pbm) or a CHIKV containing, artificial bloodmeal (titer in the bloodmeal: 10^7^ pfu/mL) at 8, 24, 32, 48, 72, and 96 h post-infectious bloodmeal (pibm). The midgut tissue of sugarfed females appears relaxed and deeply invaginated. Midgut-associated circular and longitudinal muscles are visible. Red arrows point to slight tissue damage occasionally visible, due to tearing of the basal lamina (BL). At 8 h pibm, the outer layer of the BL is torn and disrupted in close proximity to muscle tissue (exemplified by white arrows). Disruption of the outer over-stretched BL layer adjacent to muscle tissue is strongly apparent at 24 pbm/pibm and 32 h pibm. At 48 h pibm, the midgut BL is beginning to relax and contract, torn areas of the BL near to muscles become less prominent. This trend continues; at 72–96 h pibm, disrupted regions of the outer BL layer are still visible although less prominent. Images were generated using a FEI Quanta 600F scanning electron microscope. All images (except: 24 h pbm at 2753× magnification) were captured at 5000× magnification. BL = basal lamina; Mu = muscle; TC = tracheal cell. Additional (replicate) images are presented in Figure S1.

Ultrastructural imaging of the midgut surface during bloodmeal digestion via SEM revealed the overstretching of the BL surrounding the bloodfed, and/or CHIKV-infected midgut (Figure 2, Figure S1). The midgut surface structure of sugarfed mosquitoes appeared relaxed and consisted of deep invaginations with clusters of epithelial cells bulging out from the surface. As indicated by the red arrows in Figure 2 and Figure S1, occasional slight tears in the BL were visible, which were not as prominent and regularly apparent as in the bloodfed midgut samples (exemplified by white arrows in Figure 2). At 8 h post-infectious bloodmeal (pibm), the BL was thinly stretched and its outmost layer(s) was (were) torn near to the circular and longitudinal muscles surrounding the midgut organ. Between 24 and 48 h pibm, BL overstretching and tearing near muscles was strongly apparent in all samples that had been analyzed. From 72 h pibm onwards, the midgut tissue became increasingly relaxed and extensively invaginated. Areas where the BL was torn became less prominent, but were still visible at 96 h pibm. All of the SEM images showing midgut tissue after bloodfeeding were obtained from mosquitoes that also had been orally infected with CHIKV (with the exception of 24 h pbm in Figure 2). The virus titer in the bloodmeal was ~10^7^ pfu/mL. Although tiny globular structures that resembled virions were occasionally apparent on midgut surfaces, we were not sufficiently convinced that these structures were indeed virions. Multiple attempts to conduct immuno-gold labeling on these samples failed due to technical difficulties. To overcome this shortcoming, FIB-SEM tomography was conducted by collecting and processing 108 consecutive images, each 20 nm apart, of virus infected midgut epithelium. As described in the next paragraph, this approach enabled us to detect and visualize CHIK virions within the BL structure.

### 3.2. CHIKV Disseminates from the Midgut within a Narrow Time Window during Bloodmeal Digestion

**Figure 3:**
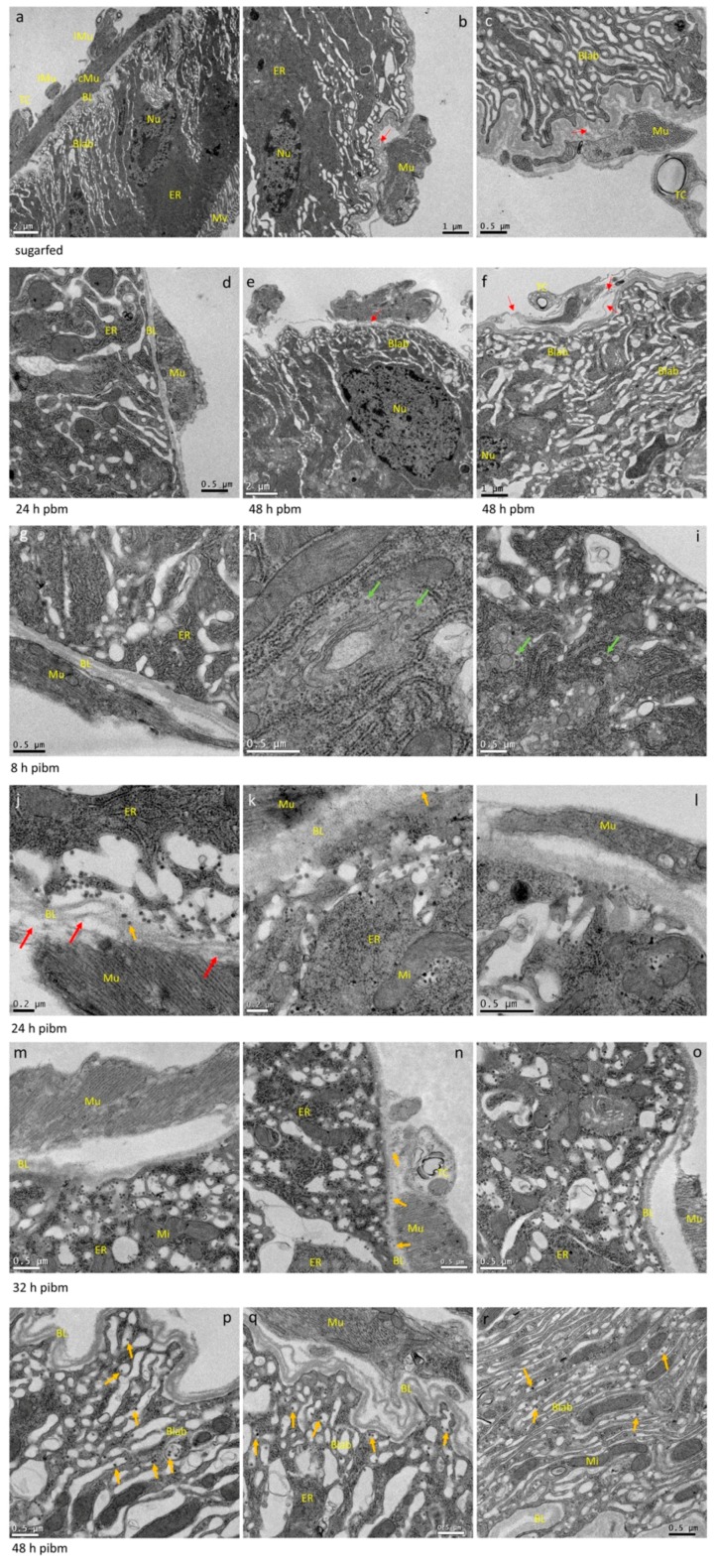
Ultrastructural (TEM) imaging of midgut cross-sections obtained from female *Ae. aegypti*, which had been sugarfed, bloodfed or orally infected with CHIKV (titer in the bloodmeal: 10^7^ pfu/mL). (**a**–**c**) midgut cross sections of sugarfed mosquitoes. The midgut epithelium consists of a single cell layer. Luminal microvilli of an epithelial cell are visible in (**a**), followed by a dense ER containing zone, the nucleus, and a prominent basal labyrinth, which stretches out until reaching the BL surrounding the entire organ. At the hemocoel facing side of the BL (outside the midgut), circular and longitudinal muscles, as well as tracheal cells are visible. In sugarfed midguts, the BL appears relaxed although splicing of individual strands is occasionally visible (red arrows). (**d**–**f**) The BL of bloodmeal containing midguts is stretched and in proximity to muscles and tracheal cells, BL strands are often severely spliced (red arrows). (**g**–**i**) At 8 h post-infectious bloodmeal (pibm), mature CHIK virions close to the BL are not yet apparent. Instead, in the ER, groups of particles resembling CHIKV nucleocapsids (40 nm diameter) are visible within membranous compartments (green arrows). (**j**–**l**) Predominantly in zones around the muscles, mature CHIK virions are concentrated within the cavities of the basal labyrinth and strongly accumulating at the BL at 24 h pibm; virions are occasionally visible within strands of the BL (orange arrows). In the zones next to muscles, the BL is distorted as strands are severely spliced (red arrows) and/or the overall texture of the BL looks porous. (**m**–**o**) At 32 h pibm, the situation described for 24 h pibm has further developed as mature CHIK virions are now more frequently observed within strands of the BL (orange arrows) indicative of virus dissemination from the midgut. (**p**–**r**) At 48 h pibm, the midgut BL looks wavy, due to progressive bloodmeal digestion causing the midgut tissue to contract. As shown in (**p**,**q**), BL strands look severely spliced, especially in proximity to muscle tissue. CHIK virions no longer accumulate in large quantities at the BL. Instead, they are dispersed and solitary within the cavities of the basal labyrinth (exemplified by orange arrows). This situation prevails at later time points, for example at 5 days pibm (see also Figure 6a,b) until the mosquito ingests a second bloodmeal. (**a**–**r**) Three different midgut samples per time point were analyzed. Images were generated using a JEOL JEM 1400 transmission electron microscope. Magnifications ranged from 2500× to 10,000×. BL = basal lamina; Blab = basal labyrinth; ER = endoplasmic reticulum; lMu = longitudinal muscle; cMu = circular muscle; Mu = muscle; Mi = mitochondrion; Mv = microvilli; Nu = nucleus; TC = tracheal cell.

TEM visualization of a posterior midgut cross section shows the organization of the single cell layer. At the luminal side, the cell layer possesses microvilli for nutrient absorption followed by a zone largely comprised of ER, nucleus, Golgi-apparatus, and mitochondria, which is interspersed with another zone defined as the basal labyrinth (Figure 3a–c and Figure S2), [8]. Virion assembly and maturation of alphaviruses take place at the plasma membrane within the basal labyrinth [61], which stretches out to the BL surrounding the midgut and separating it from the hemocoel. 

**Figure 4:**
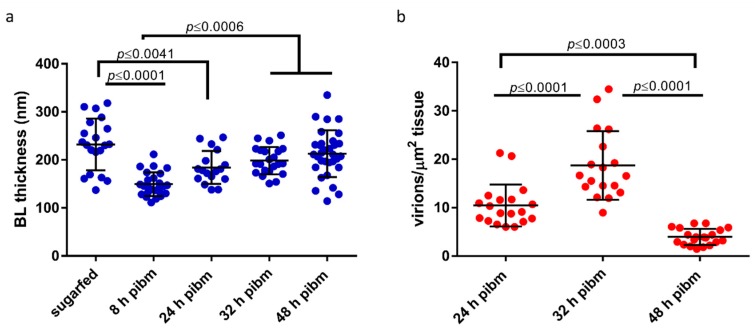
(**a**) Thickness of the midgut basal lamina (BL) of *Ae. aegypti* and (**b**) CHIK virion density in the basal labyrinth of infected midgut epithelial cells at different time points post-infectious bloodmeal (pibm). (**a**) Measurements were performed in those regions where the BL looked intact and was not severely spliced. (**a**,**b**) Each data point represents a single distinctive TEM image. Bars indicate mean values, Error bars represent standard deviations. Significant differences between mean values based on One-way ANOVA followed by Tukey’s multiple comparisons test are shown.

In our cross-sections, the principal organization of the midgut epithelium of a sugarfed mosquito looked similar to that of bloodfed mosquitoes at 24 or 48 h pbm (Figure 3a–f). However, in midguts of sugarfed mosquitoes, the BL was more relaxed and wavy in appearance, whereas in the bloodfed midguts, especially before 48 h pbm, the BL was stretched out. Individual measurements of BL thickness confirmed that in sugarfed mosquitoes the midgut BL was on average 232 nm thick (*N* = 20), whereas at 8 h pibm, the BL was on average only 149 nm thick (*N* = 25) (Figure 4a). 

Concurrent with progressive bloodmeal digestion, the average BL thickness gradually increased overtime to reach between 32 h and 48 h pibm a thickness similar to that of non-bloodfed (sugarfed) mosquitoes. Predominantly near to muscles, the BL was distorted and spliced into its individual strands as indicated by the red arrows in Figure 3. This was observed in midguts of bloodfed and to a lesser extent also in those of sugarfed mosquitoes. At 8 h pibm (titer in the bloodmeal: ~10^7^ pfu/mL, which is within the range of typical human viremia titers [62]), mature CHIK virions were not yet visible in the basal labyrinth. Instead, *de novo* assembled capsids were occasionally visible within vesicle-like structures in the cytoplasm (Figure 3h–i, green arrows). These virus-like structures had a diameter of around 40 nm, resembling the size of assembled alphavirus capsids [19,63] and were absent in non-infected control samples (Figure 3a–f). Sixteen hours later (at 24 h pibm), however, mature virions were apparent in the basal labyrinth in close proximity to the BL (Figure 3j–l and Figure S2). A few virions were visible between strands of the BL (orange arrows). In general, the virus was unevenly distributed throughout the epithelium of a midgut with typically only 2–3 regions (cells) showing strong virus infection. Accordingly, the average virion density was ~10 virions/μm^2^ (Figure 4b). We consistently noticed that the prominent regions where virions tended to amass were those in close proximity to circular and longitudinal muscles surrounding the midgut. As mentioned and shown above, these were also the regions where the multilayered BL appeared to be spliced into individual strands (red arrows). We assume that these distorted, spliced BL regions corresponded to the torn areas of BL that were visible in the SEM preparations (Figure 2 and Figure S1). 

**Figure 5:**
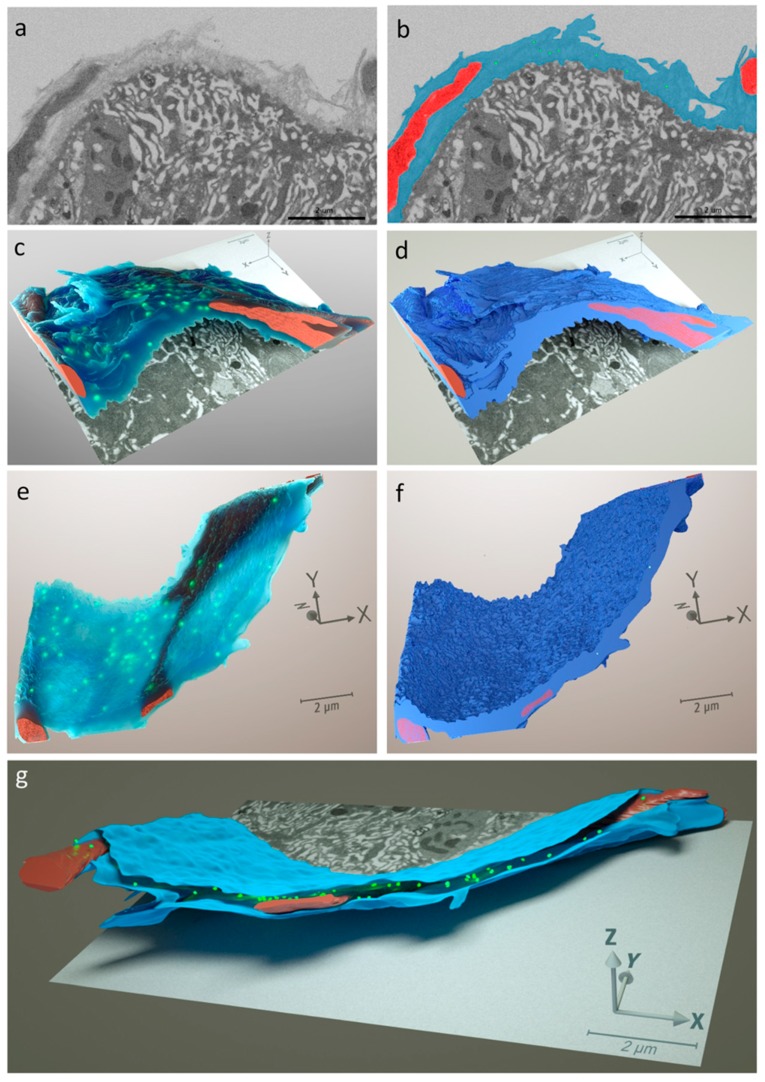
Three-dimensional (3-D) reconstruction from 108 stacked FIB-SEM-generated images serially sectioned at 20-nm intervals showing midgut tissue obtained from a CHIKV infected mosquito at 24 h post-infectious bloodmeal (pibm). (**a**) Two-dimensional (x, y) sample image (# 101) out of a stack of 108 FIB-SEM images (z = 20 nm) showing image resolution before image alignment and rendering of the three-dimensional model. (**b**) Same image as shown in (**a**) with false coloration of muscles (red), BL (cyan) and CHIK virions (green). (**c**) Global, translucent view of the sample section that had been analyzed by FIB-SEM following three-dimensional modeling. Blue coloration: Midgut BL (note the disrupted outer layer(s) of the BL); red coloration: Muscle tissue surrounding the midgut. CHIK virions (green) are clearly visible being embedded within the multi-layered BL. The basal labyrinth of the epithelial cell is visible along the x and y axes. (**d**) Opaque representation of the same sample shown in panel c. CHIK virions are absent on the BL surface facing the hemocoel. (**e**) Another 3-D view of the translucent midgut BL structure showing the presence of virions within the BL layers. (**f**) Those virions are no longer visible in the opaque (surface) representation of the same sample. (**g**) Frontal view presenting the innermost (top) and outermost (bottom) layers of the midgut BL with individual CHIKV virions being located between those BL layers. All image reconstructions were initially generated using the Amira software suite v. 5.3.1. Images were then further refined in Maxon Cinema 4D R19 using the software OTOY Octane Render V3. Bars in (**a**–**g**) represent a length of 2 μm.

Our FIB-SEM analysis based on serial imaging at 20 nm intervals confirmed that the BL formed a multi-layered dense mesh surrounding the mosquito midgut and that CHIKV was associated with the BL (Figure 5a–g). At 24 h pibm, CHIK virions were detected within the three-dimensional space between the layers of the BL when segmented and rendered as a translucent structure (Figure 5c,e). Regions of the BL in close proximity to muscles appeared to be distorted (Figure 5c,d,g). In general, virions within the BL were individually dispersed rather than densely clustered together. However, when the BL was presented as an opaque structure, virions were not visible on the BL surface, but within the inner- and outermost layers of the BL (Figure 5d,f,g). It may be possible that any virions located on the BL surface have been washed away during FIB-SEM sample processing. 

**Figure 6:**
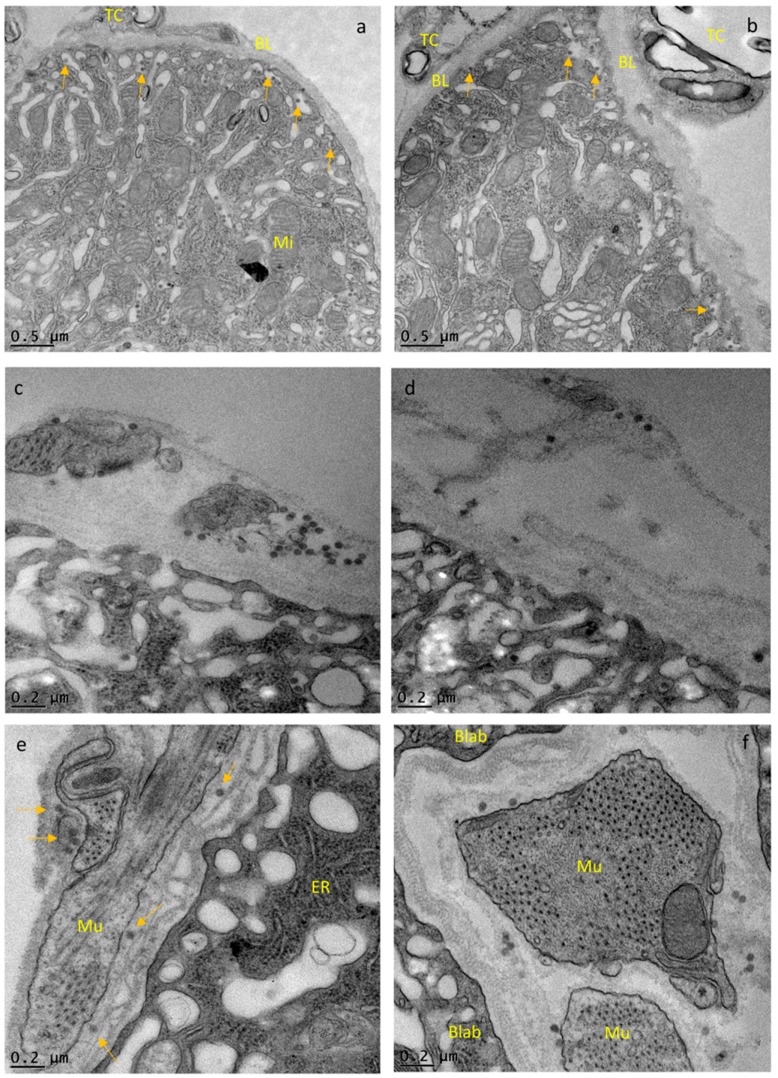
Ultrastructural (TEM) examination to assess how multiple bloodmeals affect CHIKV dissemination from the midgut of *Ae. aegypti*. Midgut cross-sections of females that had received either a single CHIKV containing bloodmeal (titer in the bloodmeal: 10^7^ pfu/mL) or an initial bloodmeal containing CHIKV followed by a non-infectious (NI) bloodmeal were analyzed. (**a**,**b**) Mature CHIK virions are dispersed throughout the cavities of the basal labyrinth of the infected midgut epithelial cell at 5 days post-initial, infectious bloodmeal. Occasionally, single virions are visible close to the BL (orange arrows). (**c**,**d**) At 24 h post-second (NI) bloodmeal (144 h post-initial CHIKV containing bloodmeal), virions are found within strands of the BL and attached to the side of the BL facing the hemocoel suggesting that these virions have traversed the BL. Occasionally, virions are also found to be attached to BL fragments that are largely detached from the main BL surrounding the midgut organ. (**e**,**f**) At 32 h post-second (NI) bloodmeal (152 h post-initial CHIKV containing bloodmeal), virions are still present between strands of the BL or associated with the hemocoel facing side of the BL, predominantly in proximity to the muscle tissue (orange arrows). (**a**–**f**) Images were generated using a JEOL JEM 1400 transmission electron microscope. Magnifications ranged from 2500× to 25,000×. BL = basal lamina; Blab = basal labyrinth; ER = endoplasmic reticulum; Mu = muscle; Mi = mitochondrion; Nu = nucleus; TC = tracheal cell.

At 32 h pibm, virion accumulation at the BL was further enhanced (average virion density: ~19 virions/μm^2^; Figure 4b), with CHIK virions in the TEM preparations now being more frequently visible between strands of the BL (Figure 3m–o, orange arrows; Figure S2). Importantly, we did not observe CHIKV infecting the midgut associated muscles at any time point during the 48 h observation period. We also did not detect virions in the tracheal tissue surrounding the midgut in ~30 different samples that were analyzed, with a single exception at 40 h pibm (Figure S3a,b, orange arrows). At 48 h pibm, the midgut epithelium became less stretched as indicated by the wavy shape of the BL (Figure 3p–r). Furthermore, virions no longer amassed towards the BL. Instead, they became more dispersed inside the basal labyrinth (average virion density: ~4 virions/μm^2^; Figure 4b) as exemplified by orange arrows in Figure 3p–r. At later time points, i.e., 5 and 7 days pibm, this pattern still prevailed (Figure 6a,b), [8]. Based on these observations, we propose that there is a relatively narrow time span from approximately 24 h to less than 48 h pibm, during which CHIKV has the opportunity to exit the midgut epithelium across the BL to infect secondary tissues. 

### 3.3. Repeated Bloodmeals Enhance Virion Dissemination from the Midgut Epithelium

We exposed mosquitoes that had been orally infected with CHIKV via an initial bloodmeal to a second, non-infectious (NI) bloodmeal (at 5 days post-initial bloodmeal) to see whether the midgut dissemination process we had observed after ingestion of a single bloodmeal was repeated during a subsequent bloodmeal digestion. At 5 days post-single infectious bloodmeal, virions were dispersed throughout the cavities of the basal labyrinth in proximity to the BL. At this time point virions did not amass at the BL (Figure 6a,b, orange arrows). However, at 24 and 32 h post second NI bloodmeal, CHIK virions were repeatedly observed outside the midgut epithelium in association with strands of the BL (Figure 6c–f). This differs from the observations made in midguts of those mosquitoes, which had received only a single, infectious bloodmeal. As described above, in singly bloodfed females, CHIK virions were observed between strands of the BL, but not outside the BL. 

### 3.4. Quantification of Alphavirus RNA in Infected Midguts and Carcasses over Time Indicates that Virus Dissemination from the Midgut Occurs within 48 h pibm

**Figure 7:**
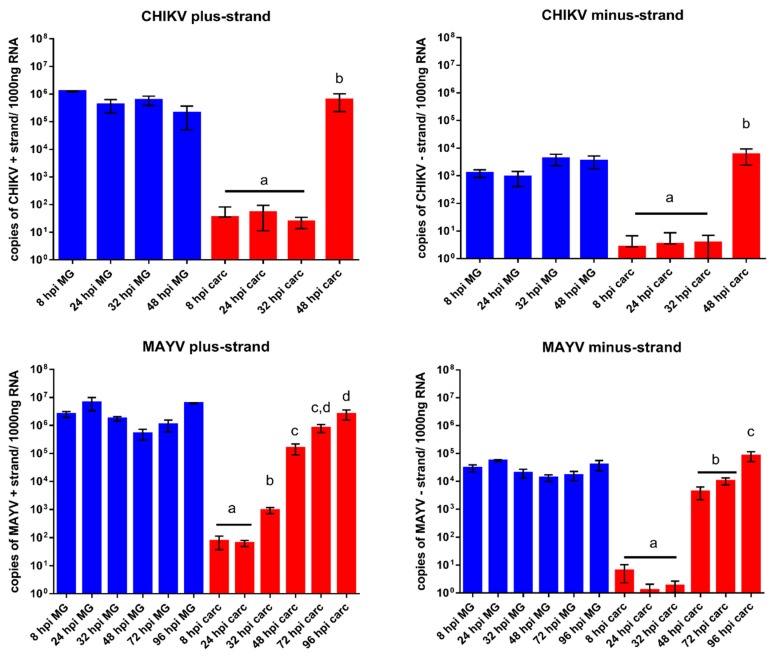
Quantitative detection of plus- and minus-strand RNAs of CHIKV and Mayaro virus (MAYV) via Taqman qRT-PCR assays in midguts (blue) and carcasses (red) of *Ae. aegypti* at various time points post-infectious bloodmeal. Each sample consisted of total RNA extracted from 10 midguts or three carcasses of CHIKV or MAYV challenged mosquitoes. For each time point the mean value of three biological replicates (=three samples) is shown. Error bars represent standard deviations. Different letters above bars (shown only for carcass values) indicate significant differences of mean values based on One-way ANOVA followed by Tukey’s multiple comparisons test (*p* < 0.05).

When comparing the viral replication of CHIKV (strain 37997) with that of a related alphavirus, MAYV (strain TRVL 4675), in midguts and carcasses of HWE mosquitoes by Taqman qRT-PCR, similar patterns became apparent. In midgut tissue, quantities of plus- and minus-strand viral RNAs of both viruses reached a plateau by 8 h pibm (Figure 7). The values of viral plus-strand RNA copy number equivalents were between 10^5^ and 10^6^ for CHIKV and 10^6^ to 10^7^ for MAYV. At 8–24 h pibm, plus-strand RNA detected in midgut tissue likely includes the viral RNA of the input virus from the bloodmeal. Quantities of minus-strand RNAs, indicative of active viral replication, were 2–3 logs lower in midgut tissue than those of viral plus-strand RNA molecules. This observation is in line with the alphavirus replication strategy described above, in which viral plus-strand RNA synthesis continues throughout the infection cycle while minus-strand RNA synthesis has already been shut off [9,13]. In carcass tissue, plus- and minus-strand RNA copy number equivalents were only minimally detectable (<100 copies) for both viruses until 48 h pibm, when minus-strand RNA copy number equivalents significantly increased. This significant increase in minus-strand RNA copy number equivalents indicates productive virus infection of secondary tissues outside the midgut. We assume that there is a time gap between virus dissemination from the midgut and actual productive (measurable) infection of secondary tissues. Thus, our Taqman qRT-PCR results support our ultrastructural observations (Figure 3, Figure 4 and Figure 7) suggesting that CHIKV dissemination from the midgut occurred within 48 h pibm, before virions inside the epithelial cells stopped congregating towards the midgut BL. 

## 4. Discussion

**Figure 8:**
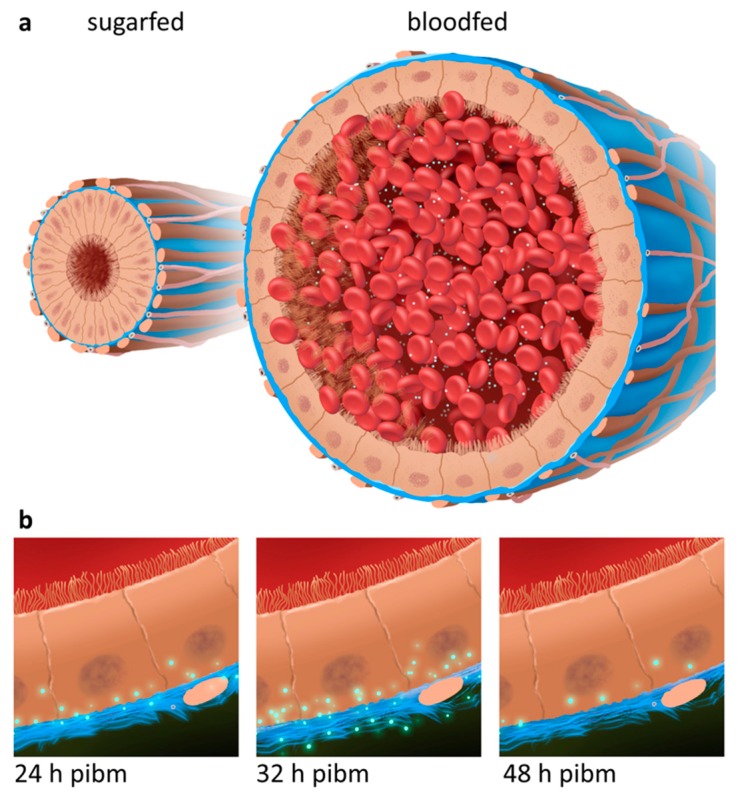
Schematic representation of CHIKV dissemination from the midgut of *Ae. aegypti*. (**a**) Midgut cross section of a sugarfed female (left) in comparison to a midgut of a female that has ingested a CHIKV-containing bloodmeal (right; virions are shown in green). Note the ~20× increase in diameter of the bloodmeal containing midgut (see also Figure 1). Also: The midgut epithelial cells of the sugarfed mosquito have a columnar shape whereas those of the bloodfed female look flattened. (**b**) Dissemination pattern of CHIKV (green) across the midgut BL (blue) at 24, 32, and 48 h post-infectious bloodmeal (pibm) according to the observations shown in Figure 3 indicating that within a time span of 48 h pibm the virus is exiting the midgut. The figure was created in Adobe Photoshop CC 2018 (Adobe Systems, San Jose, CA, USA).

In a previous study two *Ae. aegypti* laboratory strains, HWE and ORL, were analyzed for their vector competence for CHIKV (strain: 37997) [57]. The data revealed that as early as 1–2 days post-oral acquisition, the virus already produced high titers in midgut tissue and disseminated from the midgut to secondary tissues. By 48 h pibm, CHIKV was detectable in salivary glands and in saliva. These characteristics, high virus titers and fast dissemination, made CHIKV 37997 an attractive model for observing its dissemination from the midgut in a time course study at the ultrastructural level. Our artificial feeding system may not exactly reflect the amount of virus intake as might be observed with viremic bloodmeals obtained from naturally CHIKV infected hosts. Viremic bloodmeals may be smaller than artificial bloodmeals obtained via membrane feeding in a controlled environment. Furthermore, mosquitoes, such as *Ae. aegypti*, tend to ingest multiple viremic and/or non-viremic bloodmeals from the human host, which likely affect midgut infection and dissemination dynamics of an arbovirus [64,65]. However, our artificial bloodmeal procedure has the advantages of standardization and reproducibility. Earlier, we revealed that a single one-week old female ingests ~20,000 pfu CHIKV 37997 from an artificial bloodmeal containing ~10^7^ pfu/mL virus [57], a titer that is within the range of viremic bloodmeals [62]. Thus, the bloodmeal intake of a fully engorged female would amount to a volume of ~2 μL. All observations made in this study are based on these parameters. Our SEM analysis revealed that bloodmeal ingestion leads to midgut tissue (over)stretching and partial BL disruption (see also Figure 8a).

Importantly, the disruption of the BL was predominantly observed alongside muscles surrounding the midgut as the bloodmeal containing tissue was bulging out between those muscles. During progressive digestion of the bloodmeal, the midgut tissue subsequently relaxed and the damage to the BL was less pronounced although ruptures to the BL still prevailed at 72 or 96 h pibm, when the bloodmeal had been completely digested. The SEM samples included bloodfed midguts from CHIKV infected mosquitoes, however, we were unable to clearly identify midgut associated virions in those samples based on their size, shape, and quantity. We chose FIB-SEM [50,51,52,53,54] as an alternative technique and this approach enabled us for the first time to visualize virions in the three-dimensional space of a mosquito tissue. As CHIKV infection foci were irregularly distributed in the complex midgut tissue, it was initially challenging to select a suitable region of the tissue (indicated by a high abundance of virions) for FIB-SEM preparation. To overcome this complication, we prescreened a sample via TEM to identify a landmark, such as virion abundance, in a location of the sample before orienting the FIB-SEM sample for imaging. The preselected (landmarked) tissue was then mounted with the block face perpendicular to the stub surface instead of mounting it parallel to the surface of an SEM stub as a typical protocol would recommend [59,60]. This provided several advantages, including a proper orientation of the sample to the SEM beam for final data collection, a reduction in the time needed to properly expose the surface of interest using ion milling, and an easy identification of the region-of-interest using the landmark(s) established with TEM.

Our TEM images unequivocally showed the presence of CHIK virions in the midgut epithelium without any need for antibody labeling. Whereas at 8 h pibm, assembled virions were not yet visible, they became apparent in large quantities in the midgut labyrinth and in association with the BL between 24 and 32 h pibm, indicative of dissemination from the midgut (see also Figure 8b). A similar time window of virion accumulation towards the BL had been earlier reported for Western equine encephalitis virus (WEEV; *Togaviridae*, *Alphavirus*) [19,20]. Furthermore, we noticed that preferred zones of virion congregation towards the BL occurred in close proximity to muscles although muscle tissue itself did not become infected with the virus during the time period of dissemination. Our TEM images also showed that in close proximity to muscles, the BL strands often looked severely spliced and distorted corresponding to the zones of disrupted BL seen in the SEM and FIB-SEM images. However, we did not find any indication that the BL developed visible, distinct gaps through which virions could move. Instead, the movement through the BL resembled a migration/penetration-like process through the overall stretched-out BL and this process might have been be assisted by extracellular matrix associated proteinases, such as MMPs, as suggested earlier [18,49]. Our observations are partially in agreement with those made by Romoser and colleagues [21,66] regarding the dissemination of RVFV from the midgut of *Ae. taeniorhynchus*. The authors reported that the BL had a modified, “porous, spongy” structure in close proximity to midgut-associated muscles, which appeared to be permissive for virions whereas the intact (non-modified) BL was not. Further, the authors suggested that arboviruses, such as RVFV, would penetrate the modified BL to infect the tracheo-muscular complex as a conduit for dissemination from the midgut. In our studies, we did not reveal that CHIKV would infect the tracheo-muscular complex as an essential step in the dissemination process from the midgut. Instead, our ultrastructural observations based on TEM and FIB-SEM suggest that within 24–48 h pibm, CHIK virions directly traverse the BL in order to infect secondary tissues outside the midgut (Figure 8b). Accordingly, our Taqman qRT-PCR assays showed significantly increased replication of both CHIKV and MAYV in tissues outside the midgut at 48 h pibm. This similar timing for both viruses is remarkable, since the 37997 strain of CHIKV is a highly-passaged, laboratory adapted strain, whereas the TRVL 4675 (type) strain of MAYV from 1954 has a low passage history [57,58].

The relatively short time span between the first appearance of virus-like structures in the ER of the midgut epithelial cells and that of mature virions close to the BL suggests that CHIKV requires only a single replication cycle in the midgut epithelium before being able to disseminate to secondary tissues during bloodmeal digestion. However, following initial infection, virion production in the midgut epithelium continues and is independent of virus dissemination as shown by the fact that infection foci in the midgut tissue do increase over time [46,57]. We previously described that there was only a weak correlation between CHIKV titers in the midgut and the virus’ ability to disseminate from the midgut [57]. Based on our observations, we speculate that not every mature virion localized in the basal labyrinth might escape the midgut when the BL is permissive during bloodmeal digestion. Those virions “left behind” may get another opportunity for dissemination during a subsequent bloodmeal without the need of an additional replication step. When another NI bloodmeal had been ingested by the CHIKV-infected female at 120 h post-initial infectious bloodmeal, virions again strongly congregated at the stretched BL (at 24–32 h post second NI bloodmeal). Furthermore, virions were associated with the BL at its hemocoel facing side suggesting that these virions had actually traversed the BL. As pointed out above, it is possible that CHIK virions that remained within the basal labyrinth after the initial bloodmeal digestion are those that predominantly disseminated from the midgut during second bloodmeal digestion. Further, we speculate that during the BL rebuilding process after initial bloodmeal digestion, spliced fragments of the BL could have occasionally been shed from the remaining BL and in some instances, virions could have been attached to these shed BL fragments. 

Immunohistochemical and ultrastructural studies have previously shown that the tracheal system of the mosquito can be infected with a range of arboviruses, including CHIKV [15,19,21,41,42,57,66,67]. Furthermore, several studies suggested that the tracheal system acts as a conduit for virus dissemination from the midgut [21,41,66]. Salazar and colleagues [41] reported dengue 2 virus (*Flaviviridae*) infection of midgut associated tracheae as soon as 2 days pibm. At that time point the virus was also detectable in the mosquito carcass. Thus, it cannot be ruled out that other tissues outside the midgut were already infected by the disseminated virus before it infected the midgut associated tracheae. Dong and colleagues [57] showed that CHIKV was already detectable outside the midgut before the virus was located in tracheal tissue, supporting the idea of tracheal cells becoming infected after virus dissemination from the midgut. After analyzing approximately 30 different TEM samples of various time points post-CHIKV infection, we detected only a single incidence where virions were associated with a tracheal cell. Based on the singular observation here and our earlier confocal microscopy analysis [57], we do not consider this tissue to play a critical role in the midgut dissemination process of CHIKV. 

The role of apoptosis in viral midgut escape has not been clearly revealed so far and therefore remains elusive [68,69,70,71]. Previously, we have shown that bloodmeal ingestion can trigger apoptosis in midgut epithelial cells although the presence of CHIKV in the bloodmeal did not have any measurable effect on the apoptotic response in midgut tissue [57]. In this study, we did not find any evidence that CHIKV is causing any obvious pathology in the epithelial cells of the mosquito midgut. Occasionally, we detected a degenerating cell suggestively undergoing apoptosis without any virus being present [31], (Figure S3c). Furthermore, the BL surrounding the degenerating cell looked intact, allowing the conclusion that an apoptotic response in a cell may not necessarily affect the integrity of the surrounding BL. This in turn would not create any benefit for CHIKV regarding its dissemination from the midgut. This is in contrast to earlier studies with Eastern equine encephalitis virus and WEEV in *Cu. melanura* and *Cx. tarsalis*, wherein severe virus-associated pathology was observed in infected midgut tissue [31,32]. Perhaps these observations resembled extreme situations based on particular virus strain-mosquito strain combinations, since we did not observe such severe responses associated with CHIKV infection of *Ae. aegypti*. 

In summary, our study suggests that following a single replication cycle in the midgut epithelium, CHIK virions disseminate during bloodmeal digestion from the midgut epithelium by traversing the (over-) stretched BL surrounding the midgut. The virus thereby is not infecting the muscle tissue and does not use the tracheal cell system to disseminate from the midgut. An important point that requires further investigation is the underlying mechanism causing the BL to become permissive for the virus. Recent research work suggested that there may be an enzymatic BL modification/remodeling process involved that would include the activity of extracellular proteases [18,49]. Furthermore, it needs to be investigated whether other arboviruses, such as members of the *Flaviviridae* or *Bunyaviridae*, utilize the same midgut escape strategy as has been observed for CHIKV in this study. 

## Supplementary Materials

The following are available online at http://www.mdpi.com/1999-4915/10/10/571/s1, Figure S1: Ultrastructural (SEM) imaging of the midgut surface of sugarfed and bloodfed (CHIKV-infected) *Ae. aegypti*, Figure S2: Higher magnification of ultrastructural (TEM) images showing midgut cross-sections obtained from female *Ae. aegypti*, Figure S3: Rare events observed during the ultrastructural TEM time course study, Table S1: Oligonucleotide primers used for Taqman qRT-PCR to detect CHIKV and MAYV cDNAs, Table S2: Sizes and volumes of midguts obtained from sugarfed and bloodfed *Ae. aegypti*.
