# Supplementary material for: Ultrastructural Analysis of Chikungunya Virus Dissemination from the Midgut of the Yellow Fever Mosquito, Aedes aegypti
Source: Viruses. 2018 Oct 18;10(10):571. doi: 10.3390/v10100571 (PMC6213114; doi:10.3390/v10100571)
Supplement: Supplementary file 1 [file viruses-10-00571-s001.zip › viruses-368690-supplementary.pptx]

## Slide 1
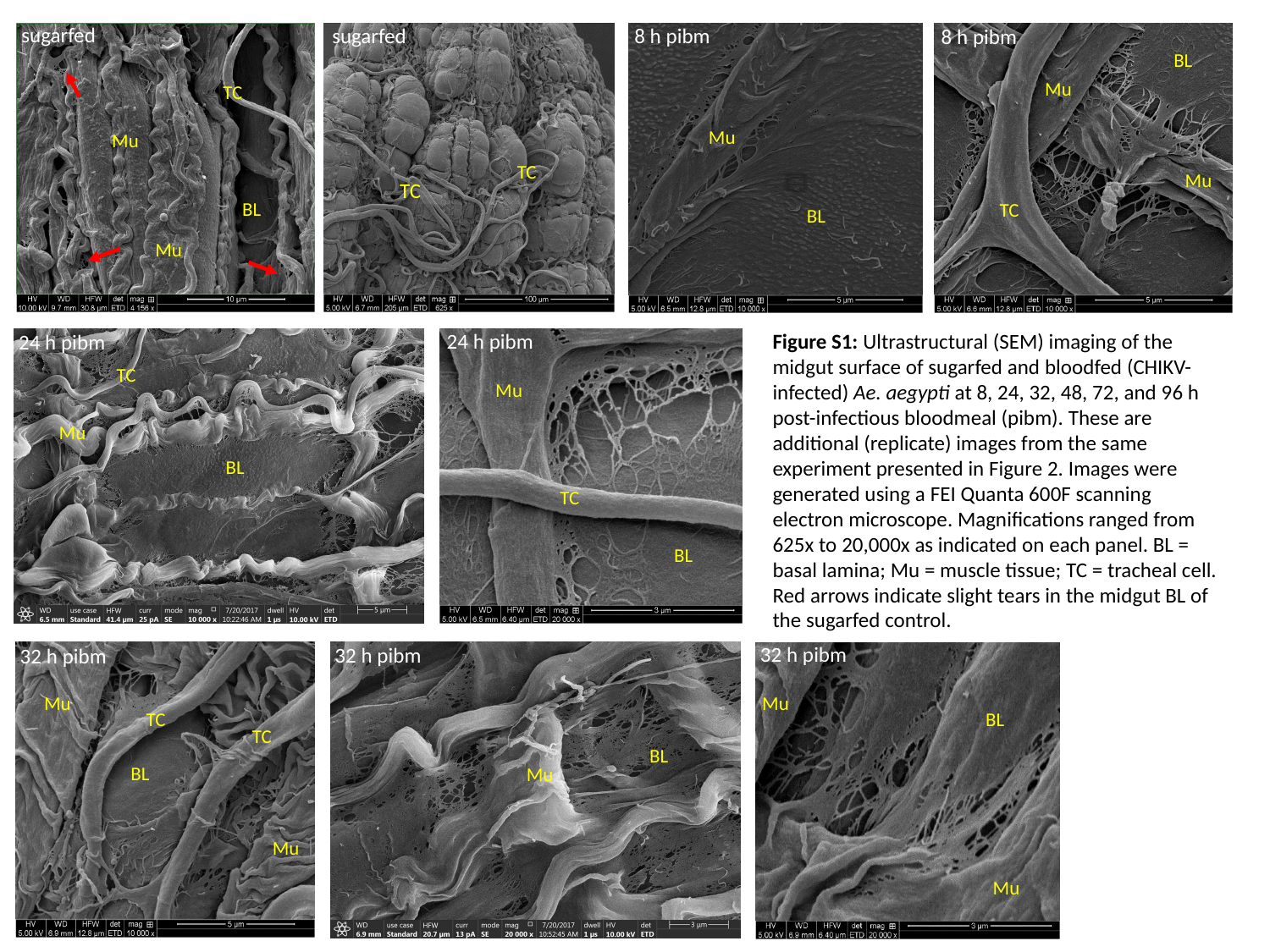

sugarfed
sugarfed
8 h pibm
8 h pibm
c
Mu
BL
BL
Mu
Mu
TC
TC
Mu
TC
TC
BL
Mu
24 h pibm
Figure S1: Ultrastructural (SEM) imaging of the midgut surface of sugarfed and bloodfed (CHIKV-infected) Ae. aegypti at 8, 24, 32, 48, 72, and 96 h post-infectious bloodmeal (pibm). These are additional (replicate) images from the same experiment presented in Figure 2. Images were generated using a FEI Quanta 600F scanning electron microscope. Magnifications ranged from 625x to 20,000x as indicated on each panel. BL = basal lamina; Mu = muscle tissue; TC = tracheal cell. Red arrows indicate slight tears in the midgut BL of the sugarfed control.
24 h pibm
TC
Mu
BL
Mu
TC
BL
g
32 h pibm
32 h pibm
32 h pibm
Mu
TC
TC
BL
Mu
Mu
BL
Mu
h
k
BL
Mu

## Slide 2
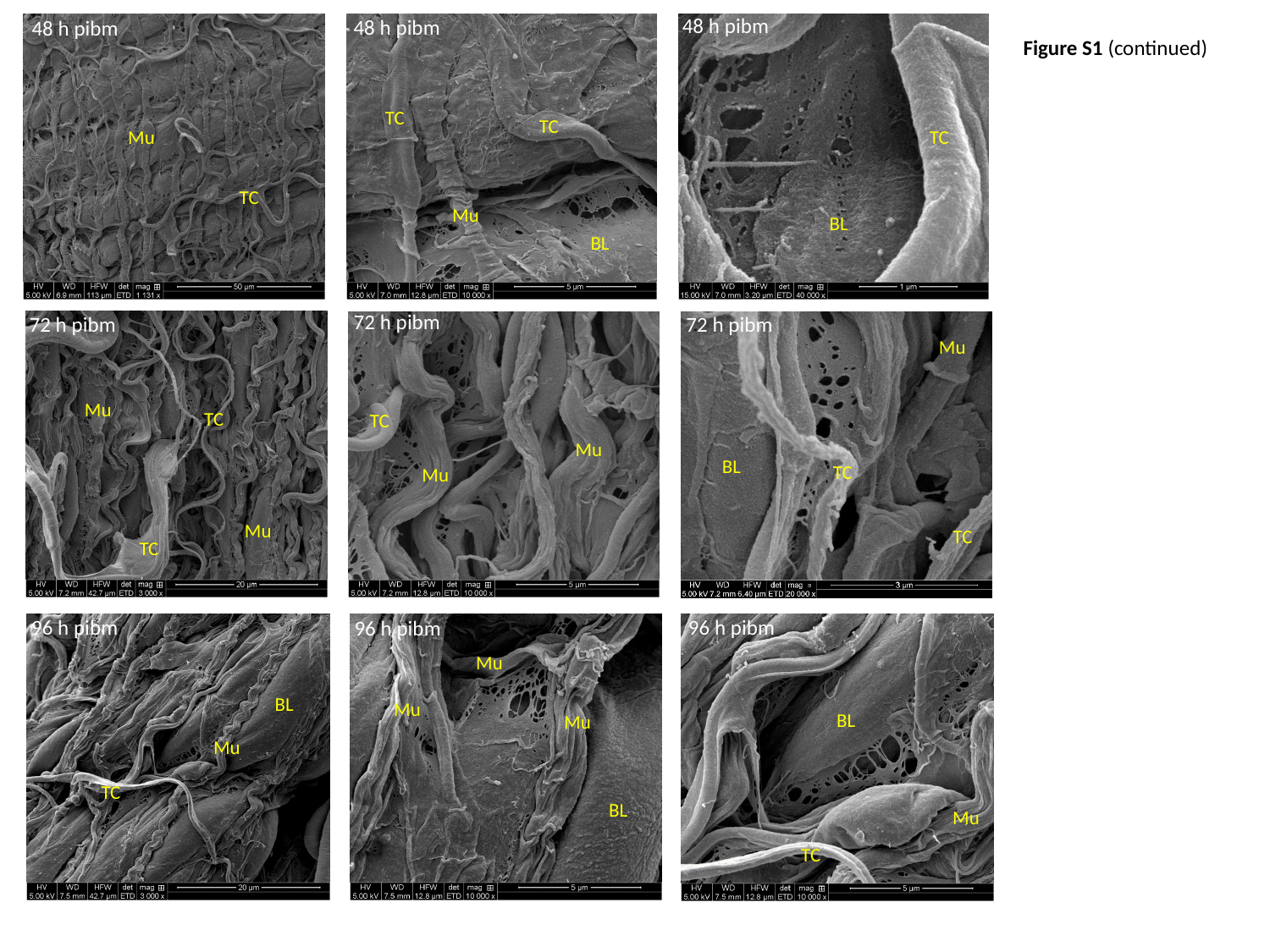

48 h pibm
48 h pibm
48 h pibm
Mu
TC
TC
TC
Mu
BL
TC
BL
Figure S1 (continued)
72 h pibm
72 h pibm
72 h pibm
Mu
TC
Mu
TC
TC
Mu
Mu
Mu
BL
TC
TC
96 h pibm
96 h pibm
96 h pibm
BL
Mu
TC
Mu
Mu
Mu
BL
BL
Mu
TC

## Slide 3
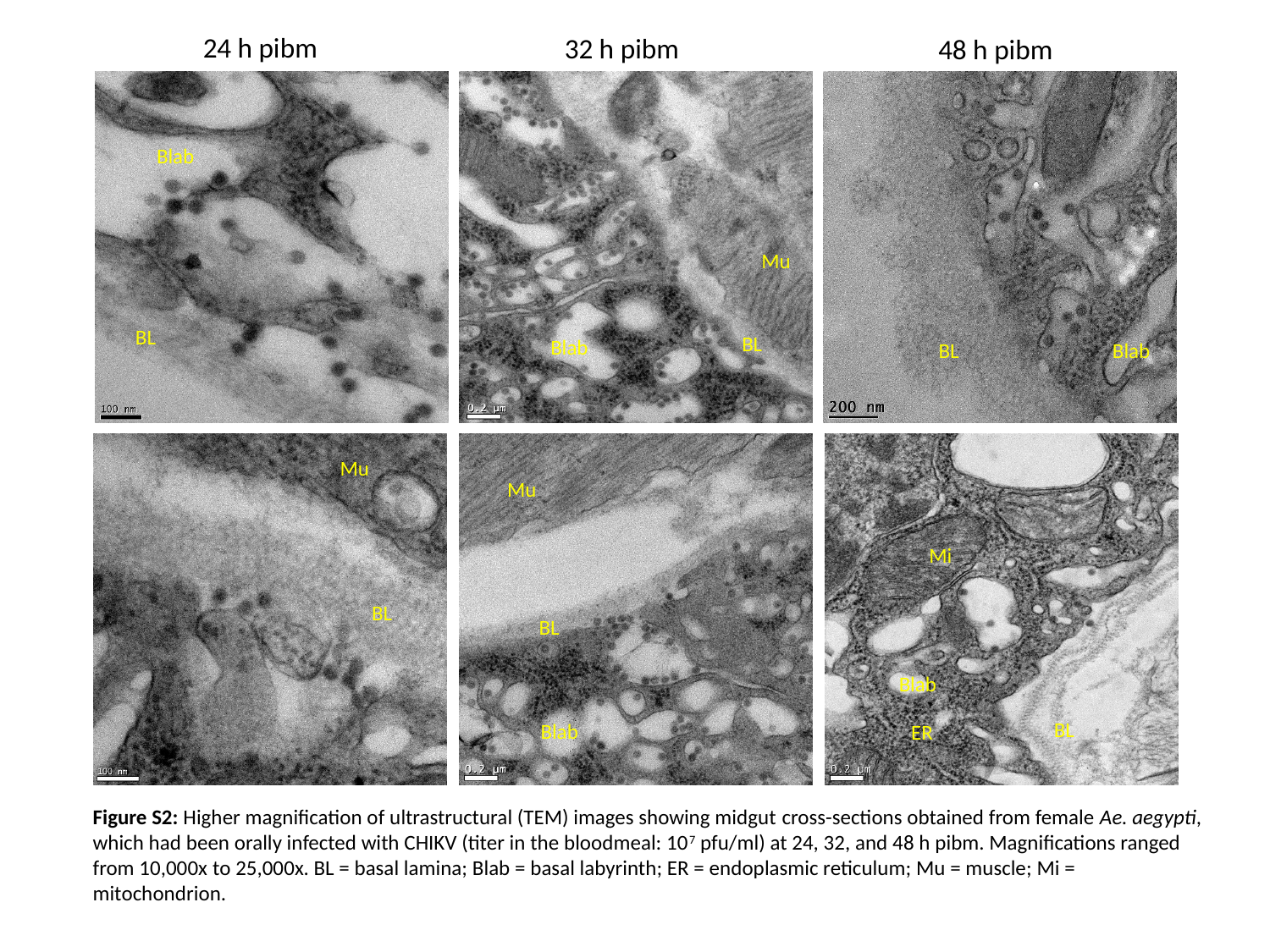

24 h pibm
32 h pibm
48 h pibm
Blab
BL
Mu
BL
Blab
Blab
BL
Mu
BL
Mu
BL
Blab
Mi
Blab
BL
ER
Figure S2: Higher magnification of ultrastructural (TEM) images showing midgut cross-sections obtained from female Ae. aegypti, which had been orally infected with CHIKV (titer in the bloodmeal: 107 pfu/ml) at 24, 32, and 48 h pibm. Magnifications ranged from 10,000x to 25,000x. BL = basal lamina; Blab = basal labyrinth; ER = endoplasmic reticulum; Mu = muscle; Mi = mitochondrion.

## Slide 4
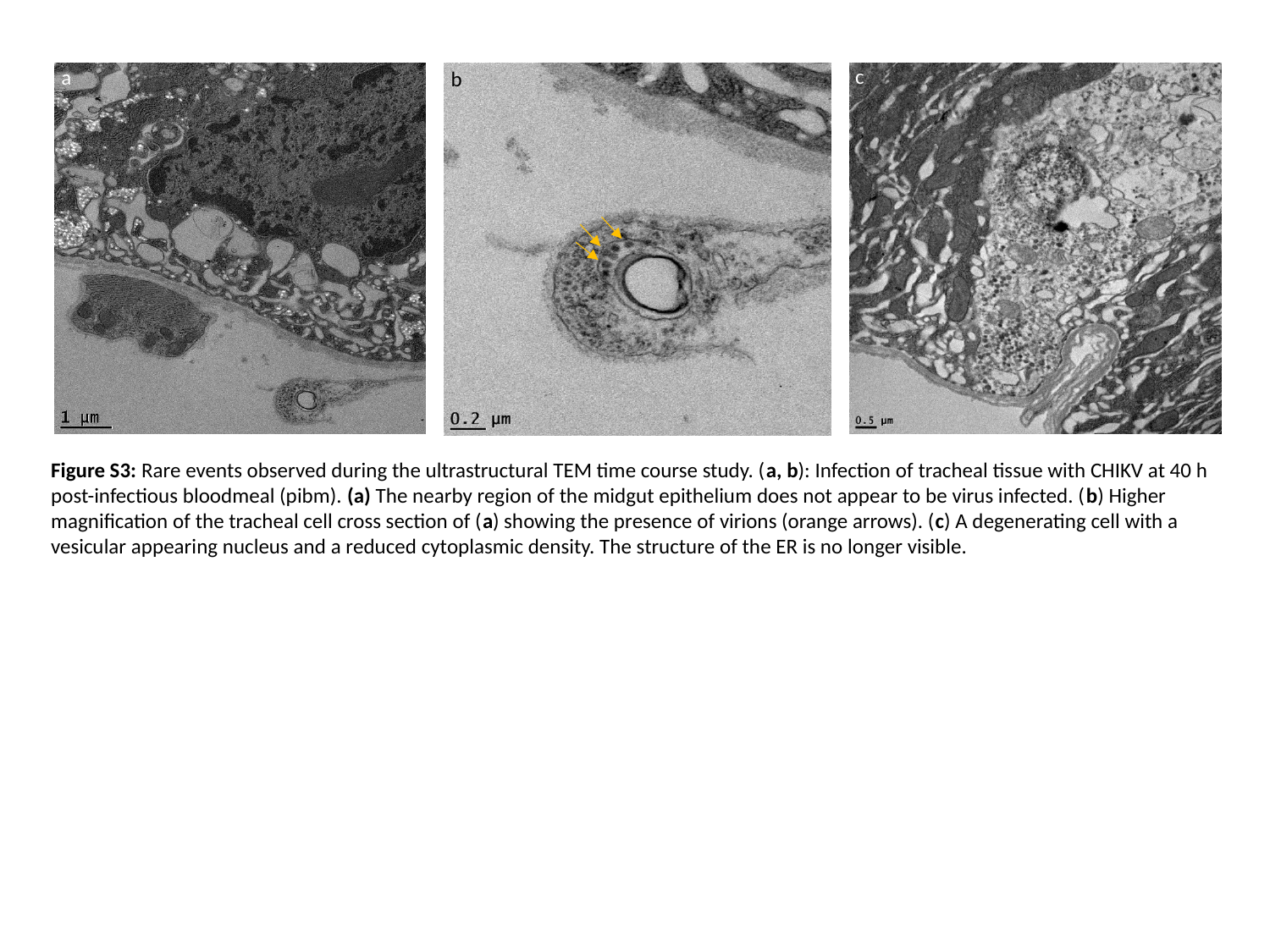

c
a
b
Figure S3: Rare events observed during the ultrastructural TEM time course study. (a, b): Infection of tracheal tissue with CHIKV at 40 h post-infectious bloodmeal (pibm). (a) The nearby region of the midgut epithelium does not appear to be virus infected. (b) Higher magnification of the tracheal cell cross section of (a) showing the presence of virions (orange arrows). (c) A degenerating cell with a vesicular appearing nucleus and a reduced cytoplasmic density. The structure of the ER is no longer visible.

## Slide 5
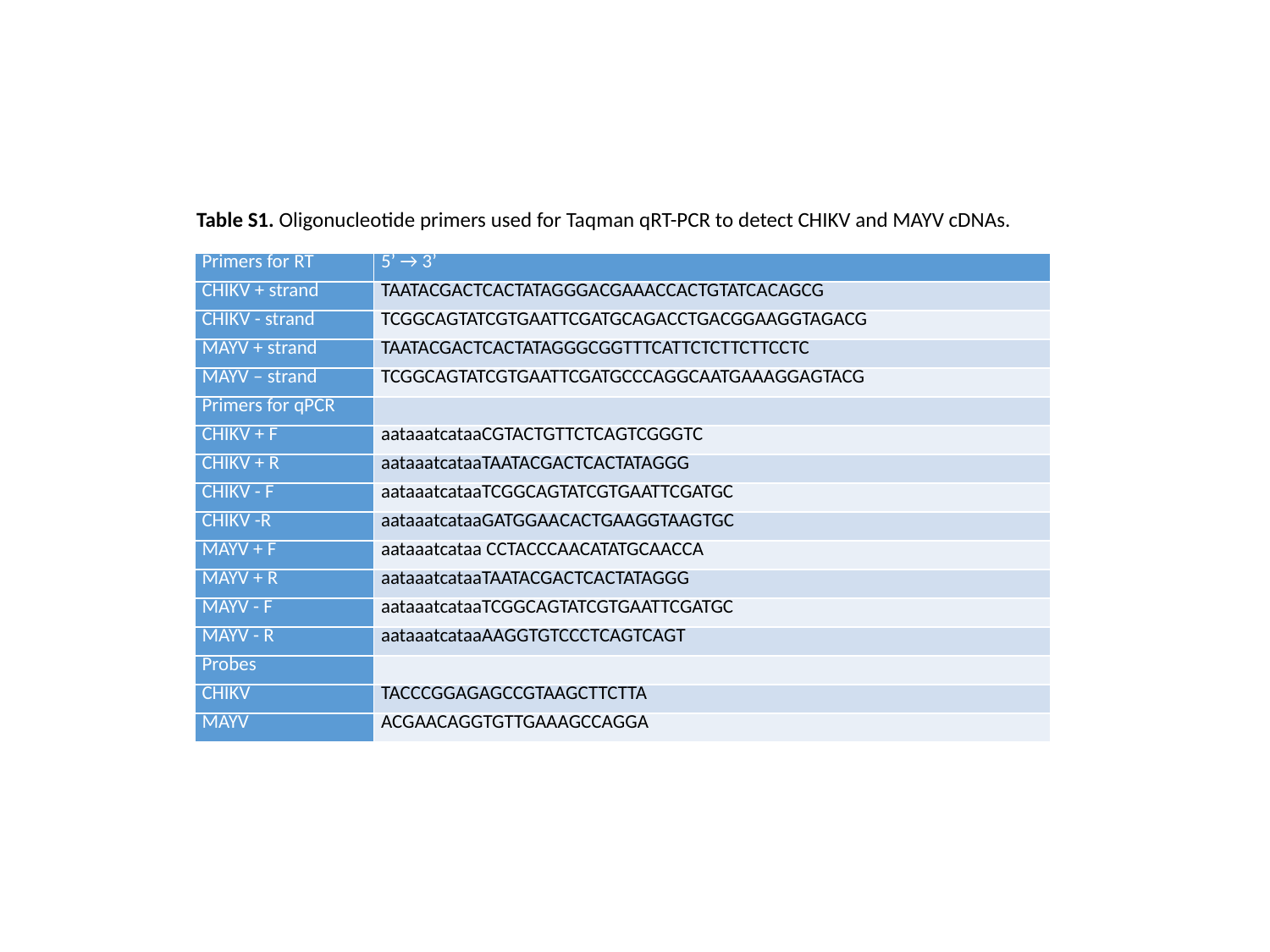

Table S1. Oligonucleotide primers used for Taqman qRT-PCR to detect CHIKV and MAYV cDNAs.
| Primers for RT | 5’ → 3’ |
| --- | --- |
| CHIKV + strand | TAATACGACTCACTATAGGGACGAAACCACTGTATCACAGCG |
| CHIKV - strand | TCGGCAGTATCGTGAATTCGATGCAGACCTGACGGAAGGTAGACG |
| MAYV + strand | TAATACGACTCACTATAGGGCGGTTTCATTCTCTTCTTCCTC |
| MAYV – strand | TCGGCAGTATCGTGAATTCGATGCCCAGGCAATGAAAGGAGTACG |
| Primers for qPCR | |
| CHIKV + F | aataaatcataaCGTACTGTTCTCAGTCGGGTC |
| CHIKV + R | aataaatcataaTAATACGACTCACTATAGGG |
| CHIKV - F | aataaatcataaTCGGCAGTATCGTGAATTCGATGC |
| CHIKV -R | aataaatcataaGATGGAACACTGAAGGTAAGTGC |
| MAYV + F | aataaatcataa CCTACCCAACATATGCAACCA |
| MAYV + R | aataaatcataaTAATACGACTCACTATAGGG |
| MAYV - F | aataaatcataaTCGGCAGTATCGTGAATTCGATGC |
| MAYV - R | aataaatcataaAAGGTGTCCCTCAGTCAGT |
| Probes | |
| CHIKV | TACCCGGAGAGCCGTAAGCTTCTTA |
| MAYV | ACGAACAGGTGTTGAAAGCCAGGA |

## Slide 6
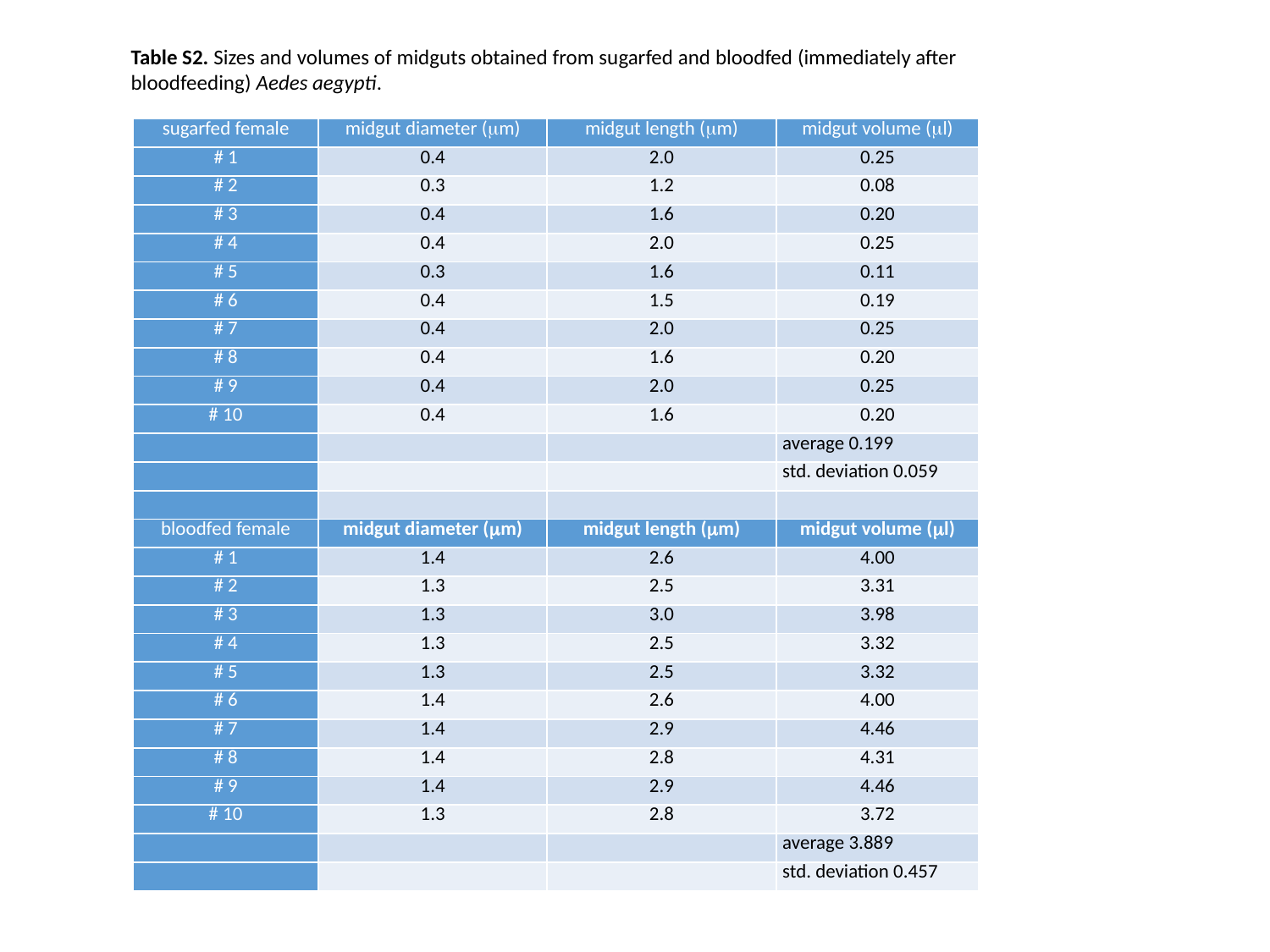

Table S2. Sizes and volumes of midguts obtained from sugarfed and bloodfed (immediately after bloodfeeding) Aedes aegypti.
| sugarfed female | midgut diameter (mm) | midgut length (mm) | midgut volume (ml) |
| --- | --- | --- | --- |
| # 1 | 0.4 | 2.0 | 0.25 |
| # 2 | 0.3 | 1.2 | 0.08 |
| # 3 | 0.4 | 1.6 | 0.20 |
| # 4 | 0.4 | 2.0 | 0.25 |
| # 5 | 0.3 | 1.6 | 0.11 |
| # 6 | 0.4 | 1.5 | 0.19 |
| # 7 | 0.4 | 2.0 | 0.25 |
| # 8 | 0.4 | 1.6 | 0.20 |
| # 9 | 0.4 | 2.0 | 0.25 |
| # 10 | 0.4 | 1.6 | 0.20 |
| | | | average 0.199 |
| | | | std. deviation 0.059 |
| | | | |
| bloodfed female | midgut diameter (mm) | midgut length (mm) | midgut volume (ml) |
| # 1 | 1.4 | 2.6 | 4.00 |
| # 2 | 1.3 | 2.5 | 3.31 |
| # 3 | 1.3 | 3.0 | 3.98 |
| # 4 | 1.3 | 2.5 | 3.32 |
| # 5 | 1.3 | 2.5 | 3.32 |
| # 6 | 1.4 | 2.6 | 4.00 |
| # 7 | 1.4 | 2.9 | 4.46 |
| # 8 | 1.4 | 2.8 | 4.31 |
| # 9 | 1.4 | 2.9 | 4.46 |
| # 10 | 1.3 | 2.8 | 3.72 |
| | | | average 3.889 |
| | | | std. deviation 0.457 |
